# Bioactivities of Mealworm (*Alphitobius diaperinus* L.) Larvae Hydrolysates Obtained from Artichoke (*Cynara scolymus* L.) Proteases

**DOI:** 10.3390/biology11050631

**Published:** 2022-04-20

**Authors:** Luis Tejada, Laura Buendía-Moreno, Irene Hernández, Adela Abellán, José María Cayuela, Eva Salazar, Estefanía Bueno-Gavilá

**Affiliations:** Department of Human Nutrition and Food Technology, Universidad Católica de Murcia UCAM Campus de los Jerónimos, 30107 Murcia, Spain; ltejada@ucam.edu (L.T.); irene.hernandezr9@gmail.com (I.H.); aabellan@ucam.edu (A.A.); jmcayuela@ucam.edu (J.M.C.); esalazar@ucam.edu (E.S.)

**Keywords:** *Alphitobius diaperinus* L. larvae, angiotensin-I-converting enzyme (ACE) inhibitor, antioxidant, artichoke, bioactive peptide

## Abstract

**Simple Summary:**

Edible insects can provide an alternative and sustainable source of dietary protein to meet the future demand of the growing global population. Therefore, this study analysed the impact of mealworm (*Alphitobius diaperinus* L.) larvae hydrolysis using artichoke (*Cynara scolymus* L.) flower’s enzyme extract, on the production of peptide hydrolysates with potential bioactivity. The antioxidant capacity against the 1,1,-diphenil-2-picrylhydrazyl (DPPH) radical and the angiotensin-I-converting enzyme inhibitory activity of the hydrolysates were determined. Furthermore, the identification of the peptide sequences was conducted to detect the potential bioactive peptides in the hydrolysates. The results reveal that the water-soluble extract of artichoke flower could be suitable for producing bioactive peptides from mealworm larvae, and could be incorporated as an ingredient in future functional food products.

**Abstract:**

In this study, we aimed to obtain hydrolysates with bioactive peptides from mealworm (*Alphitobius diaperinus* L.) larvae using an artichoke (*Cynara scolymus* L.) enzyme extract. Two types of substrates were used: the raw larvae flour (LF) and its protein extract (PE). The hydrolysis yield, considering the peptide concentration of the hydrolysates, was higher in PE hydrolysates than in LF hydrolysates (6.39 ± 0.59 vs. 3.02 ± 0.06 mg/mL, respectively). However, LF showed a higher antioxidant activity against the DPPH radical than PE (59.10 ± 1.42 vs. 18.79 ± 0.81 µM Trolox Eq/mg peptides, respectively). Regarding the inhibitory activity of angiotensin-I-converting enzyme (ACE), an IC_50_ value of 111.33 ± 21.3 µg peptides/mL was observed in the PE. The identification of the peptide sequence of both hydrolysates was conducted, and LF and its PE presented 404 and 116 peptides, respectively, most with low molecular weight (<3 kDa), high percentage of hydrophobic amino acids, and typical characteristics of well-known antioxidant and ACE-inhibitory peptides. Furthermore, the potential bioactivity of the sequences identified was searched in the BIOPEP database. Considering the antioxidant and ACE-inhibitory activities, LF hydrolysates contained a larger number of sequences with potential bioactivity than PE hydrolysates.

## 1. Introduction

The prevalence of diseases such as hypertension or cardiovascular problems is related to nutritional factors. This relationship between health and nutrition has given rise to a growing interest in society for functional foods, which, besides their nutritional characteristics, offer healthy properties [1]. One component of these functional foods is bioactive peptides, because they provide a beneficial physiological effect due to their multifunctional biological activities such as antioxidative, antihypertensive, antimicrobial, anticancer, antidiabetic, and immunomodulatory, among others [2]. These peptides are short chains of amino acids that are inactive within the original protein and can be released during gastrointestinal digestion or during the industrial processing of certain foods [3]. Obtaining peptides from proteins can be conducted using different methods such as microbial fermentation, used mainly for dairy products, and enzymatic hydrolysis, being the fastest and easiest technique to control [4]. Bioactive peptides are being widely studied to determine their effects on the key systems of the human body, such as the digestive, cardiovascular, nervous, and immune systems. Among the bioactive peptides, those with antihypertensive activity are probably the most studied [5]. In recent years, peptides with antihypertensive activity have been identified in dairy [6,7], egg [8], marine [9], and vegetable [10] products. Insects can also be a good source of bioactive peptides, because they have a high protein content (over 70% of dry weight), which allows the supply of essential amino acids with high nutritional and unsaturated fat levels [11], compared to the contribution from other animals for consumption [12,13]. Therefore, there is considerable interest in promoting and improving the consumption of insects, which have a growing presence in the agri-food chain, as transformed animal protein derived from insects can be used to produce feed for farm animals and aquaculture [14]. Recently, the European Union has authorized the commercialization of three insect species for human consumption: *Tenebrio molitor*, *Locusta migratoria*, and *Acheta domesticus*, that comply with the Regulation (EU) 2015/2283 on novel foods [15]. In the future, using edible insects as a functional ingredient to develop enriched foods could be considered because of the presence of bioactive peptides. However, the research of bioactive peptide extraction in insects is limited, with some reported studies on crickets, locusts, grasshoppers, and silkworms [16]. The silkworm is one of the most studied insects for obtaining bioactive peptides with antimicrobial, antihypertensive, antioxidant, antidiabetic, anticancer, and hepatoprotective properties [17,18]. Studies about antihypertensive activity in insect protein hydrolysates were published for various species (*Bombix mori*, *Bombus terrestris*, *Schistocerca gregaria*, and *Spodoptera littoralis*), using enzymes of different origin (gastrointestinal proteases, thermolysin, and alcalase), and observing inhibitory activity of the angiotensin-I-converting enzyme (ACE) [19]. Furthermore, the antioxidant activity of insect hydrolysates (*S. littoralis*, *G. sigillatus*, *A. diaperinus*, *B. mori*, *B. dubia*, etc.) has also been determined when using different enzymes, mostly gastrointestinal proteases, but also thermolysin, alcalase, and papain [20,21,22,23]. The most common way of obtaining bioactive peptides is through enzymatic hydrolysis, mainly using enzymes of animal origin; thus, it is convenient to study other proteases of plant origin such as cynarases—aspartic proteases present in flowers of the genus *Cynara* [24]. Enzyme extracts are obtained from the mature artichoke flower, *C. scolymus* L. These enzymes present a wide proteolytic activity and produce elevated concentrations of hydrophobic peptides demonstrated to possess higher bioactivity than hydrophilic ones [5,25]. Moreover, the mature artichoke flower constitutes an agricultural residue at the end of the season, and its use to obtain enzymes extracts could give rise to the revaluation of this waste culture. Thus, the aim of this study was to prepare hydrolysates from larvae of *A. diaperinus* L. with enzymatic extracts of the artichoke flower and to evaluate their antioxidant and ACE-inhibitory activities. Furthermore, the peptide sequence identification of the hydrolysates was conducted and the deliverables were analyzed using the BIOPEP database [26].

## 2. Materials and Methods

Mature artichoke flowers (*C. scolymus* L.) were collected from the Region of Murcia (Spain). Vacuum-packed *A. diaperinus* L. larvae were provided by Proti-Farma (Holland). ACE from rabbit lung, the ACE synthetic substrate, hippuryl-L-histidyl-L-leucine (HHL), and 1,1-diphenil-2-picrylhydrazyl) (DPPH) were purchased from Sigma Chemicals Co. (St. Louis, MO, USA).

### 2.1. Preparation of C. scolymus L. Extract

The stigmas and stiles of the mature artichoke flower were macerated for 24 h in distilled water at room temperature. The water-soluble extract was filtrated and then centrifuged for 5 min at 4000× *g*. The supernatant obtained was filtrated, lyophilized and kept at −20 °C until use [27]. The enzymatic extract protein concentration [28] was 0.104 ± 0.01 (*w*/*w*).

### 2.2. A. diaperinus L. larvae Flour and Protein Extract Preparation

To obtain the larvae flour (LF), 100 g of dried *A. diaperinus* L. larvae with a 58.6% protein content were minced using a grinder until a homogeneous powder was achieved. Next, the protein extract (PE) was obtained [29]: 50 g of larval meal was diluted with 500 mL of distilled water and the pH was adjusted to 8.0 with NaOH (0.1 M). After constant stirring for 30 min, the homogenate was centrifuged at 3220× *g* for 15 min. The pH of the supernatant was adjusted to 4.5 with HCl (0.1 N) to precipitate the protein. The resulting solution was centrifuged again at 3220× *g* for 15 min and the precipitate obtained was subjected to washing phases with distilled water at pH 4.5. The protein content of the PE, determined by the Kjeldahl method [30], was 31.65% ± 0.81. The preparation of the samples (LF and PE) was performed in triplicate.

### 2.3. Obtaining Hydrolysates

Hydrolysates of LF and its PE with the artichoke enzymatic extract were prepared following the method described by Timón et al. (2014) [31], with some modifications. Briefly, substrates (LF and PE) were dissolved in citrate buffer (pH 6.2), adjusting the protein content concentration to 1% (*w*/*v*). The solutions were mixed with the enzymatic extract at a final concentration of 22.1 µg of artichoke extract protein/mL of LF or PE solution, respectively, and incubated for 16 h in a shaking bath at 50 °C, according to optimal hydrolysis conditions established previously [5]. The hydrolysis was halted by raising the temperature to 100 °C for 10 min and the remaining non-hydrolyzed protein was precipitated by adjusting the pH to 4.5 with HCl (0.1 N). The samples were centrifuged at 3220× *g* for 30 min and the supernatant was collected and filtered through 0.45 µm Nylon discs. Last, the pH was adjusted to 7.0 with NaOH (0.1 M) and distributed in Falcon tubes for storage at −20 °C until use. Each type of hydrolysate was obtained in triplicate.

### 2.4. Peptide Concentration of Hydrolysates

The quantity of peptides was determined after precipitating the residual protein with 5% trichloroacetic acid (1:2, *v*/*v*) and centrifuging at 3220× *g* for 20 min. The nitrogen content in the supernatant was determined by the Kjeldahl method [30], and the peptide concentration was calculated using a conversion factor of 6.25. The peptide content determination for each type of hydrolysate was conducted in triplicate.

### 2.5. Antioxidant Activity

The antioxidant activity against 1,1-diphenyl-2-picrylhydrazyl (DPPH) radical was determined [32]. First, the hydrolysate was mixed with ethanol (1:1, *v*/*v*). Then, 125 µL of 0.02% DPPH in ethanol (*w*/*v*) were added to 1 mL of the hydrolysate solution. The reaction was maintained for 1 h at room temperature, protected from light, and after that, was centrifuged for 2 min at 10,000× *g*. The supernatant absorbance at 517 nm was measured. The radical scavenging activity (RSA) was calculated using Equation (1):

DPPH RSA % = [((A_control_ − A_blank_) − (A_sample_ − A_blank_))/(A_control_ − A_blank_)] × 100 (1)

A_control_ being the measurement of the radical with distilled water, A_sample_ the measurement of the DPPH in presence of the sample, and A_blank_ the measurement of a solution of ethanol in distilled water (1:1, *v*/*v*). The Trolox equivalent antioxidant capacity (TEAC) of the hydrolysates (µM Trolox Eq/mg peptide) was determined using the following linear equation: y = 3.4789x + 0.0289 (R^2^ = 0.997). The analyses for the different hydrolysates were performed three times.

### 2.6. Angiotensin-I-Converting Enzyme Inhibitory Activity

The ACE inhibitory activity of the hydrolysates was conducted [33]. The hydrolysates (40 µL) were incubated at 37 °C with 100 µL 5 mM HHL dissolved in 0.1 M borate buffer and 0.3 M NaCl (pH 8.3). Then, 2 mU of ACE were incorporated into the substrate. The reaction was interrupted after 30 min, adding 150 µL of 1 M HCl. The hippuric acid formed was recovered through 1000 µL of ethyl acetate and centrifuged for 10 min at 4000× *g*; the organic phase (800 µL) was collected. Ethyl acetate was eliminated by increasing the temperature to 95 °C. The resulting hippuric acid was resuspended in 1000 µL of distilled water, and the absorbance at 228 nm was measured. The ACE inhibitory activity was determined applying Equation (2):

ACE inhibitory activity (%) = ((A_control_ − A_blank_) − (A_sample_ − A_blank_))/(A_control_ − A_blank_)) × 100 (2)

A_control_ being the measurement of hippuric acid produced by the action of non-inhibited ACE, A_sample_ the measurement of the hippuric acid produced by the action of ACE with the sample, and A_blank_ the measurement of non-reacting HHL. The IC_50_ value was obtained in triplicate.

### 2.7. Peptide Sequence Identification

The identification of the sequences of the peptides of each type of hydrolysate was accomplished through nano-liquid chromatography–tandem MS analysis, previously carrying out a trypsin digestion [34]. The MS2 spectra were searched with the SEQUEST HT engine against a UniProtKB database (2019 UniProt consortium; https://www.uniprot.org, accessed on 4 May 2020). The peptides and their respective quantification were identified (with high confidence and without post-translational modifications) from each sample through the peptide spectral matches (PSM). The quantification values were normalized, paying attention to all PSM in the sample. Thus, the quantification of a single peptide was comparable between different samples. A search for each peptide identified in the BIOPEP bioactive peptide database was conducted [26]. Two different analyses were conducted: identification of biopeptides in a bioactive state in the hydrolysate; and identification of peptides with potential bioactivity, because they contain bioactive sequence fragments in their primary structure. Data analysis was performed with R (version 3.4.0; https://www.r-project.org, accessed on 1 June 2020).

### 2.8. Statistical Analysis

Statistical analysis was performed using SPSS (version 21.0, IBM Corporation, Armonk, NY, USA), and a one-way ANOVA test was also performed. The means values were considered statistically different when *p* < 0.05.

## 3. Results

### 3.1. Effect of Hydrolysis Substrate on the Peptide Concentration

The type of substrate used in the hydrolysis (LF or PE) influenced the enzymatic production of peptides. Here, enzymatic hydrolysis of PE with artichoke flower extract after 16 h yielded a higher concentration of peptides (6.39 ± 0.59 mg/mL) than the hydrolysates from LF (3.02 ± 0.06 mg/mL) (*p* = 0.001). This may be due to the plant proteases acting more efficiently on the PE proteins than on the whole LF, because there would be no interactions with other compounds.

### 3.2. Antioxidant Activity

**Figure 1:**
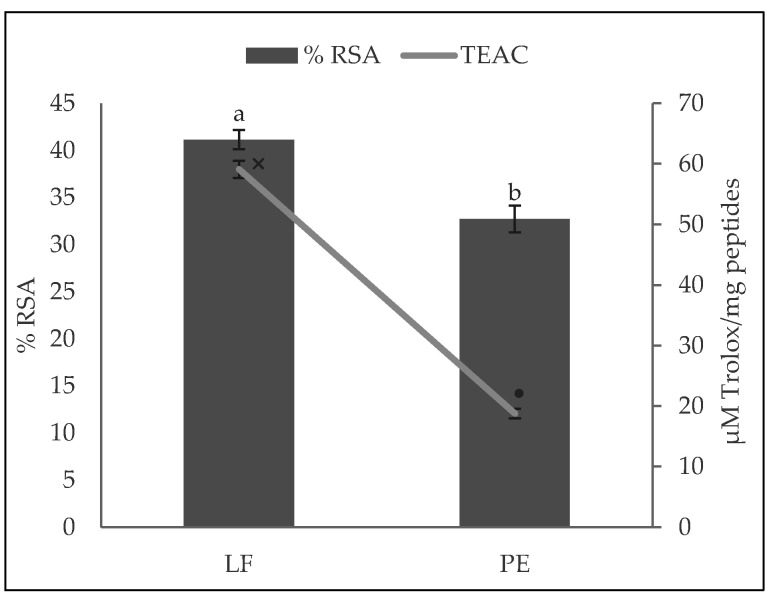
Antioxidant activity of *A. diaperinus* larvae flour (LF) and its protein extract (PE) hydrolysates against 1,1-Diphenyl-1-pycrylhydrazyl (DPPH) radical. Bars show the radical scavenging activity percentage (RSA%) and the line represents the Trolox equivalent activity capacity (TEAC: µM of Trolox equivalents per mg of peptides). Values are mean ± SD (*n* = 3). Bars with different letters (a, b) and TEAC values (line) with distinct symbols (×, •) were statistically different (*p* < 0.008 and *p* < 0.001, respectively).

The DPPH radical antioxidant activities of the LF and PE hydrolysates are presented in Figure 1. The type of substrate had a significantly statistical influence (*p* < 0.01) on the DPPH RSA of the samples. Although both hydrolysates exerted antioxidant activity, the LF hydrolysate presented a higher RSA (41.15 ± 0.99%) than PE (32.72 ± 1.41%). This difference may be due to the total LF possibly containing other compounds besides peptides, that could contribute to the antioxidant capacity, and because of the greater number of different peptides identified in this type of hydrolysate and higher quantity of potential antioxidant sequences, as described in Section 3.4.

### 3.3. ACE-Inhibitory Activity

The IC_50_ value of the ACE-inhibitory activity of the PE hydrolysates was 111.33 ± 21.3 µg peptides/mL. However, it was not possible to determine the ACE inhibition IC_50_ value of raw LF hydrolysates, because no repetitive results were obtained. This may be due to the lack of purification of this type of sample, which could cause assay interferences.

### 3.4. Peptide Sequence Identification

In LF and its PE hydrolysates, 404 and 116 peptides were identified, respectively (data not shown), of which the majority (375 and 113, respectively) had a molecular weight lower than 3 kDa. We also observed many peptides with hydrophobic residues in both hydrolysates. Thus, Table 1 and Table 2 list the LF and PE hydrolysate peptides with molecular weight <1.5 kDa that contain at least 50% of hydrophobic amino acids, respectively.

**Figure 2:**
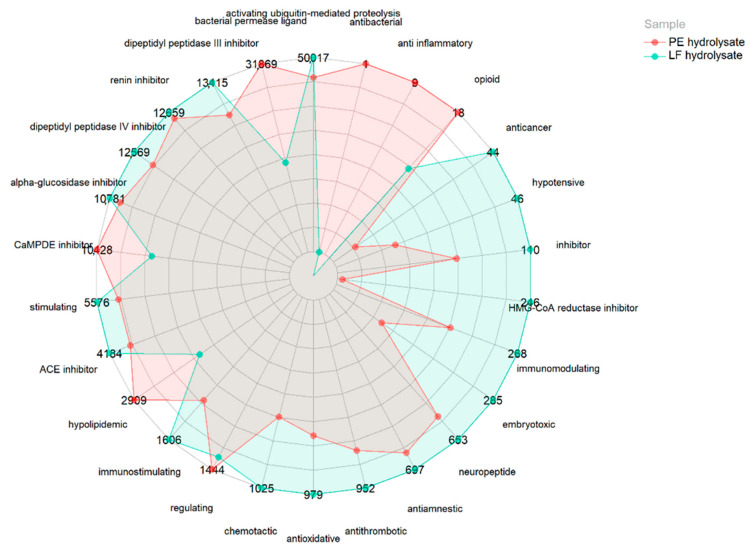
Normalized quantification of potential bioactive sequences (×10^−3^) of raw larva meal (LF) and its protein extract (PE) hydrolysates. Liquid chromatography–mass spectrometry peptide spectral matches (PSM) have been conducted considering the existence of reported bioactive sequences within their primary structure. The values were normalized considering the PSM of the total peptides in the hydrolysates. Thus, the quantification of a sequence between the hydrolysates was comparable.

No peptides were found in the bioactive peptide database (BIOPEP [21]). However, many contain fragments of sequences with reported bioactivity in their primary structure. Therefore, Figure 2 shows the distribution of a normalized quantification (based on all PSM of the peptides in the samples) of the potential bioactivities of both hydrolysates as they contain bioactive sequence fragments collected in the BIOPEP database [26]. The LF hydrolysate possessed a higher potential bioactivity because it contained a larger number of sequence fragments with previously reported bioactivity, highlighting the activities of activating ubiquitin-mediated proteolysis, bacterial permease ligand activity, dipeptidyl peptidase III inhibition, renin inhibition, dipeptidyl peptidase IV inhibition, α-glucosidase inhibition, calmodulin-dependent cyclic nucleotide phosphodiesterase inhibition (CaMPDE inhibition), stimulating activity, hypolipidemic activity, and immunostimulating activity. Moreover, the PE hydrolysate presented a greater number of potential bioactive peptide sequences with bacterial permease ligand activity, CaMPDE inhibition activity, and hypolipidemic activity. Regarding ACE inhibition, raw LF hydrolysates showed a larger number of possible sequence fragments with putative activity than PE hydrolysates (4,183,560 and 3,703,948, respectively). Likewise, considering antioxidant capacity, LF hydrolysate presented many possible sequences with bioactivity than PE hydrolysates (979,440 and 682,783, respectively).

## 4. Discussion

### 4.1. DPPH Antioxidant Activity

Studies have been conducted in which the antioxidant activity of hydrolysates from insects was also determined using in vitro techniques, with highly variable results. Vercruysse et al. determined the antioxidant activity of different cotton leafworm (*Spodoptera littoralis* L.) hydrolysates using alcalase and thermolysin enzymes, obtaining a DPPH RSA percentage lower than 20% in both cases [3]. However, Yang et al. reported a high level of DPPH RSA (67.5%) from *Bombyx mori* L. hydrolysates obtained with alcalase [22]. Furthermore, *Tenebrio molitor* hydrolysates, obtained using a combination of alcalase and flavourzyme enzymes, showed 83 µM Trolox equivalents/mg [35], which is a higher activity than that observed in *A. diaperinus* hydrolysates from artichoke extract (53.10 µM Trolox equivalents/mg). Likewise, Hall et al. reported a DPPH antioxidant capacity between 872.4 and 1490.5 µmol Trolox/mg in cricket (*Grillodes sigilatus*) protein hydrolysates using an alcalase enzyme [20].

However, artichoke enzyme extracts have been used to obtain hydrolysates with antioxidant activity from different food matrices. The antioxidant and ACE-I inhibitory activities of the hydrolysates of bovine casein, ovalbumin, and *A. diaperinus* from water-soluble enzymatic artichoke extract, over 16 h of hydrolysis, are summarized in Table 3. Thus, bovine casein and ovalbumin hydrolysates showed a DPPH antioxidant activity of 4.35 and 13.03 µM Trolox Eq/mg peptides, respectively [8,34], which is a lower activity than that observed in *A. diaperinus* LF and its PE hydrolysates (18.79 and 59.10 µM Trolox Eq/mg, respectively). These results reveal the importance of the enzyme-substrate specificity when obtaining peptides with antioxidant activity. Therefore, *A. diaperinus* protein could be considered a better hydrolysis substrate from cinarases (compared to bovine casein and ovalbumin) to obtain antioxidant peptides against the DPPH radical.

### 4.2. ACE-Inhibitory Activity

Many studies on insect bioactive peptides have evaluated their ACE inhibition capacity. Vercruysse et al. prepared different insect species protein hydrolysates (*Bombus terrestris*, *Schistocerca gregaria*, *Spodoptera littoralis*, and *Bombyx mori*) with alcalase enzyme, showing IC_50_ values of: 2.97, 19.67, 3.86, and 6.49 mg/mL, respectively. In addition, *S. littoralis* and *B. mori* thermolysin hydrolysates presented IC_50_ values of 4.54 and 1.52 mg/mL, respectively [19]. Furthermore, *S. littoralis* after digestion with gastrointestinal enzymes and mucosal peptidases showed an IC_50_ value of 0.211 mg/mL [3]. Likewise, Wu et al. obtained *B. mori* hydrolysates through simulated gastrointestinal digestion with an IC_50_ value of 1.508 mg/mL, and its 5 kDa fraction showed an IC_50_ of 0.596 mg/mL [36]. The IC_50_ values were much higher, indicating lower ACE inhibition, than observed with *A. diaperinus* PE hydrolysates here (0.111 mg/mL).

Sousa et al. prepared *A. diaperinus* hydrolysates using 2.5 L alcalase and Corolase PP. The *A. diaperinus* hydrolysate with Colorase gave an IC_50_ value of 0.107 mg/mL, which is like that observed in this study for *A. diaperinus* PE hydrolysates with artichoke enzyme extract. However, the *A. diaperinus* hydrolysate with 2.5 L alcalase showed an IC_50_ value of 0.056 mg/mL, indicating a higher ACE-inhibitory activity [37]. Furthermore, artichoke enzyme extracts have been used to obtain hydrolysates with ACE-inhibitory activity from bovine casein and ovalbumin (Table 3). Thus, the ACE inhibition of the casein hydrolysates was like that observed in *A. diaperinus* PE hydrolysates here (IC_50_ of 117.04 vs. 111.33 µg peptides/mL, respectively). However, the ovalbumin hydrolysates from artichoke extract presented a higher ACE inhibition (IC_50_: 69.55 µg peptides/mL) than given by *A. diaperinus* PE hydrolysates.

### 4.3. Identification of Bioactive Peptides

Most peptides identified in both hydrolysates had a molecular weight <3 kDa and sequence chains between 8 and 20 amino acids. Authors have reported that these peptides have a low mass, yield a greater antioxidant activity [38] and ACE inhibition, because they accommodate more efficiently to the ACE active site [39]. In addition, the bioactivity of the peptides highly depends on their sequence and amino acid composition [40]. Thus, the presence of Pro, Gly, Ala, Val, and Leu confers intrinsic antioxidant activity [41]. In both hydrolysates in this study, many peptides containing these antioxidant residues were observed, some being <1.5 kDa and having over 50% of hydrophobic amino acids (Table 1 and Table 2). Therefore, the presence of Phe, Ile, Leu, Val, Ala, and Lys amino acids at the C- and N-termini is reported to improve the radical scavenging ability of the peptides [42]. The peptides 1, 2, 5, 6, and 8–20 of the LF hydrolysate, and the peptides 1–5 of the PE hydrolysate, present these hydrophobic residues at the N-terminus and/or C-terminus. Besides, in these peptides, these hydrophobic residues are present more frequently in the C-termini, which is reported to exert a greater antioxidant effect than when they are presented in N-termini [43,44]. Moreover, bulky hydrophobic amino acids with low electronic or steric/hydrogen bonding properties, such as Trp, Tyr, Phe, Met, Leu, and Ile, at the third position next to the C-terminus, contribute to the antioxidant activity [43]. Furthermore, the presence of glutamic and aspartic acids (Glu and Asp) may be responsible for the antioxidant properties [45]. Therefore, many peptides from LF hydrolysate, such as GLIGAPIAAPI, AESEVAALNR, VDAAVLEKLEA, FSLPHAILRLDL, and YALPHAILRIDL, as well as peptides from PE hydrolysate, such as APVAVAHAAAVPA, ASVVEKLLGDY, and GLIGAPIAAPIAA, presented over one of these antioxidant characteristics. The peptide VDAAVLEKLEA of the LF hydrolysate attracted our attention, because it contains 63.64% hydrophobic amino acids, with Val and Ala at the N- and C-termini, respectively, as well as a bulky hydrophobic amino acid (Leu) at the third position next to the C-terminus, and including glutamic and aspartic acids in its sequence. Regarding these results, the peptides with these particular structures could be responsible for the antioxidant capacities of the LF and its PE hydrolysates.

Considering the in vitro ACE-inhibitory activity of the PE hydrolysate and the peptide sequences identified, it has been reported that C-terminal tripeptide residues play a predominant role in competitive binding to the active site of ACE. Studies indicated that this enzyme prefers substrates containing hydrophobic amino acid residues (aromatic or branched side chains) at each of the three C-terminal positions [1]. APVAVAHAAVPA, GLIGAPIAAPIAA, and LEKDNALDRAAM share this characteristic. Furthermore, the most effective ACE-inhibitory peptides identified contain Tyr, Phe, Trp, and/or Pro at the C-terminus [1], such as ASVVEKLGDY.

Regarding the normalized quantification of all possible bioactive sequence fragments within the primary structure of the identified peptides, the LF hydrolysate contained more sequence fragments with putative antioxidant and ACE-inhibitory activities (979,440 and 4,183,560, respectively). These results agree with the determination of antioxidant activity against DPPH in vitro in this study.

However, there are several studies of insect protein hydrolysates that have reported peptide sequences that showed bioactivity. Zhu et al., in solitary black fly (*Hermetia illucens* L.) hydrolysate with alcalase, identified several peptides (PTTAPSATIN, MAAGTNLLDTK, FPGGETEALRR, AGGGGGGGGGGGKNL, IHKAGGGGGGGGGGGK, NWDLKEVGGGALP, SATTAIYMNALL, and SLGEMKQTAK) comprising over 50% of hydrophobic amino acids which are related to high antioxidant capacity [46]. Pattarayingsakul et al. identified peptides derived from pepsin and trypsin digestion of *Oecophylla smaragdina* with high ACE-inhibitory capacity (FFGT and LSRVP) and elevated antioxidant activity (CTKKHKPNC) [47]. Furthermore, bioactive peptides were also identified from other food protein hydrolysates obtained from *C. scolymus* enzyme extract. Bueno Gavilá et al. reported 22 bioactive peptides in bovine casein hydrolysates, GPVRGPFPII; KVLPVPQK; ARHPHPKLSFM; and VKEAMAPK, with antioxidant activity, and TPVVVPPFLQP with ACE-inhibitory activity [34]. Furthermore, in ovalbumin hydrolysates from artichoke enzyme extract, peptides with antioxidant activity (IAAEVYEHTEGSTTSY and PIAAEVYEHTEGSTTSY) and with ACE-inhibitory activity (HLFGPPGKKDPV and YAEERYPIL) were reported [8].

## 5. Conclusions

In this study, *A. diaperinus* larvae hydrolysates obtained with artichoke enzyme extracts have demonstrated antioxidant and ACE-inhibitory activities in vitro. The antioxidant capacity was higher in the raw larvae hydrolysate compared to the protein extract hydrolysate, probably due to the presence of other compounds with antioxidant properties besides peptides. The analytical results agree with the potential bioactivity of the peptide sequences identified in both hydrolysates. Therefore, artichoke enzyme extract could be used to obtain multifunctional hydrolysates from insect protein that could be incorporated into future functional foods.

This study shows that an agricultural residue of great economic importance could be revalorized, providing a sustainable and more environmentally friendly source of protein. However, additional studies involving antihypertensive and antioxidant activities in vivo and potential cytotoxicity effects are required.

## Figures and Tables

**Table 1:** Amino acid sequences of peptides from raw larvae flour hydrolysate determined by nLC-MS/MS with a molecular weight <1.5 kDa and minimum 50% of hydrophobic amino acids.

No.	Peptide Sequence	Experimental Mass	Protein Source	Acc.
1	GLIGAPIAAPI	991.61	Larval cuticle protein F1	Q9TXD9
2	AYVGPDGVTY	1040.49	Cuticle protein CP14.6	Q94984
3	SIGDGIARVY	1049.56	Uncharacterized protein	−
4	TVGDGIARVY	1049.56	ATP synthase subunit alpha	Q3AHK5
5	AESEVAALNR	1058.54	Tropomyosin	P31816
			Tropomyosin-2	Q1HPQ0
6	GLIGAPIAAPIA	1062.65	Larval cuticle protein F1	Q9TXD9
7	WDDMEKIW	1121.49	Actin	P6855/Q39758
			Actin-1	P49128
			Actin-2	P10984/Q9Y707
8	VDAAVLEKLE	1127.61	Arginine kinase	P91798
9	ASVVEKLGDYL	1192.64	Profilin	P25843
10	VDAAVLEKLEA	1198.65	Arginine kinase	P91798
11	AGFAGDDAPRAVF	1292.62	Actin	P68555
			Actin-2	P10984/Q9Y707
12	GLIGAPIAAPIAAPL	1343.82	Larval cuticle protein F1	Q9TXD9
13	ASLEAEAKGKAEAL	1386.74	Myosin heavy chain, muscle	P05661
14	AIANAAEKKQKAF	1388.78	Myosin heavy chain, muscle	P05661
15	FSLPHAILRLDL	1393.82	Actin-2	Q9Y707
16	YALPHAILRIDL	1393.82	Actin	P68555
			Actin-1	P49128
			Actin-2	P10984
17	VDAAVLEKLEAGF	1402.74	Arginine kinase	P91798
18	GLIGAPIAAPIAAPLA	1414.86	Larval cuticle protein F1	Q9TXD9
19	PADTPEVAAAKVAHA	1446.75	Cuticle protein 18.7	P82165
20	LKVDDLAAELDASQ	1486.76	Myosin heavy chain, muscle	P05661

**Table 2:** Amino acid sequences of peptides from larvae protein extract hydrolysate determined by nLC-MS/MS with a molecular weight <1.5 kDa and minimum 50% hydrophobic amino acids.

No.	Peptide Sequence	Experimental Mass	Protein Source	Acc.
1	APVAVAHAAVPA	1072.61	Cuticle protein 38	P04375
2	VAYSPAAVVSH	1099.57	Larval/pupal cuticle protein H1C	P80686
3	ASVVEKLGDY	1079.56	Profilin	P25843
4	GLIGAPIAAPIAA	1133.69	Larval cuticle protein F1	Q9TXD9
5	LEKDNALDRAAM	1361.67	Tropomyosin-2	Q1HPQ0

**Table 3:** Antioxidant activity against DPPH radical and ACE-I inhibitory activity of different protein substrate hydrolysates (bovine casein and ovalbumin) obtained with water-soluble enzymatic artichoke extract in comparison with the *A. diaperinus* total larvae flour (LF) and its protein extract (PE) hydrolysates.

Protein Substrate	ACE-I Inhibitory Activity	TEAC against DPPH Radical
	(IC_50_: µg Peptides/mL)	(µM Trolox/mg Peptides)
*A. diaperinus* LF	−	59.10 ± 1.4
*A. diaperinus* PE	111.33 ± 21.3	18.79 ± 0.8
Bovine casein [34]	117.04 ± 3.3	4.35 ± 0.7
Ovalbumin [8]	69.55 ± 3.1	13.03 ± 0.4

## Data Availability

Data sharing not applicable.
